# Highly Durable Antimicrobial Tantalum Nitride/Copper Coatings on Stainless Steel Deposited by Pulsed Magnetron Sputtering

**DOI:** 10.3390/mi13091411

**Published:** 2022-08-27

**Authors:** Thangavel Elangovan, Athinarayanan Balasankar, Selvaraj Arokiyaraj, Ramaseshan Rajagopalan, Rani P. George, Tae Hwan Oh, Parasuraman Kuppusami, Subramaniyan Ramasundaram

**Affiliations:** 1 Smart Energy Materials Research Lab (SEMRL), Department of Energy Science, Periyar University, Salem 636011, India; 2 Department of Physics, Gobi Arts & Science College, Gobichettipalayam 638476, India; 3 Department of Food Science & Biotechnology, Sejong University, Seoul 05006, Korea; 4 Materials Science Division, Indira Gandhi Centre for Atomic Research, Kalpakkam 603102, India; 5 Department of Nanoscience and Nanotechnology, Bharathiar University, Coimbatore 641046, India; 6 School of Chemical Engineering, Yeungnam University, Gyeongsan 38436, Korea; 7 Center for Nanoscience and Nanotechnology, Sathyabama Institute of Science and Technology, Chennai 600119, Tamilnadu, India

**Keywords:** antibacterial activity, tantalum nitride, copper, nanocomposite, coating, magnetron sputtering

## Abstract

Highly durable and antimicrobial tantalum nitride/copper (TaN/Cu) nanocomposite coatings were deposited on D-9 stainless steel substrates by pulsed magnetron sputtering. The Cu content in the coating was varied in the range of 1.42–35.42 atomic % (at.%). The coatings were characterized by electron probe microanalyzer, X-ray diffraction, scanning electron microscope and atomic force microscope. The antibacterial properties of the TaN/Cu coatings against gram-negative *Pseudomonas aeruginosa* were evaluated using a cell culture test. The peak hardness and Young’s modulus of TaN/Cu with 10.46 at.% Cu were 24 and 295 GPa, respectively, which amounted to 15 and 41.67% higher than Cu-free TaN. Among all, TaN/Cu with 10.46 at.% exhibited the lowest friction coefficient. The TaN/Cu coatings exhibited significantly higher antibacterial activity than Cu-free TaN against *Pseudomonas aeruginosa*. On TaN, the bacterial count was about 4 × 10^6^ CFU, whereas it was dropped to 1.2 × 10^2^ CFU in case of TaN/Cu with 10.46 at.% Cu. The bacterial count was decreased from 9 to 6 when the Cu content increased from 25.54 to 30.04 at.%. Live bacterial cells were observed in the SEM images of TaN, and dead cells were found on TaN/Cu. Overall, TaN/Cu with 10.46 at.% Cu was found to be a potential coating composition in terms of higher antimicrobial activity and mechanical durability.

## 1. Introduction

Antibacterial coatings on inanimate surfaces prevent the spread of pathogenic microbes through human contact. The threat posed by the recent COVID-19 outbreak increased the widespread use of antimicrobial coating. Particularly, commonly used metal surfaces such as stainless steel (SS) necessitate the need to develop an effective antibacterial coating [1,2,3,4]. Among numerous types of bacteria, *Pseudomonas aeruginosa (P. aeruginosa)* is a particularly pervasive bacterium that can survive on inanimate surfaces for 6 h to 16 months. Infection of this bacterium by human contact with contaminated objects can cause detrimental effects including fatalities of patients with low immunity to germs. Thus, antimicrobial coatings that can disinfect *P. aeruginosa* are essential for preventing the infections associated with hospitals and other healthcare facilities [5,6,7].

To mitigate the spread of bacteria through surface contact, numerous antibacterial coatings based on copper (Cu) and silver (Ag) have been developed [8,9,10,11]. Wan et al. [12] reported that implantation of Cu and Ag ions improved the antibacterial activity of 317L SS, titanium-aluminum-niobium alloys and titanium surfaces. Dan et al. [13] found that upon Cu ion implantation, the antibacterial property of AISI 420 SS substrates against *Escherichia coli (E. coli)* and *Staphylococcus aureus* (*S. aureus*) was enhanced. Kelly et al. [14] studied titanium nitride TiN/Ag coatings with varying Ag contents deposited on AISI 304 SS by pulsed magnetron sputtering and tested against *P. aeruginosa* and *S. aureus*. The incorporation of Ag rendered antibacterial TiN/Ag coatings with reasonable tribological properties. Cu containing TiN coatings were also fabricated on commercial SS using a hybrid dual magnetron sputtering process by Tian et al. [15]. The resultant TiN/Cu films were found to be very effective in disinfecting *E. coli*.

Elangovan et al. [16] reported that the inclusion of Cu in CrN coating on SS was effective in enhancing antibacterial activity against *P. aeruginosa*. Recently, using powder metallurgy, Liu et al. [17] prepared an antipathogen SS containing Cu and Ag. This composite was reported as useful for combating COVID-19 spread via surfaces. Hsieh et al. [18] deposited tantalum nitride (TaN)/Cu nanocomposite films on silicon wafer and M2 tool steel substrates by the combination of reactive co-sputtering and rapid thermal annealing. It was found that the antibacterial property of these coatings was substantially increased after high temperature rapid thermal annealing. Because of its covalent bonds, TaN possesses high thermal and chemical stability. Therefore, TaN has been preferred to form hard, mechanically stable, wear and corrosion resistant coatings on SS based tools and components [19]. Echavarria et al. [20] prepared Ag and Cu nanoparticle-doped TaN coatings. Among all, the antibacterial TaN/Cu coating was found to be advantageous due to its excellent anti-wear and corrosion resistant properties. However, to the best of our knowledge, adequate attention was not paid towards forming nanocrystalline TaN/Cu antimicrobial coatings on potential structural and biomedical implant material in Ti modified SS.

In the present work, antimicrobial TaN/Cu nanocomposite coatings were prepared on a titanium modified SS alloy substrate (D-9) using pulsed magnetron sputtering. The Cu composition was varied by increasing the surface area of the Cu target. The physiochemical characterization of TaN/Cu coatings was performed using X-ray diffraction, atomic force microscopy and scanning electron microscopy. Durability and wear resistance were evaluated using nano hardness analysis. The antibacterial properties were tested against *P. aeruginosa*. Cell culture, scanning electron microscopy and epifluorescence microscopy were used to determine the extent of bacterial growth and disinfection.

## 2. Materials and Methods

### 2.1. Deposition of TaN/Cu Nanocomposite on Stainless Steel

TaN/Cu nanocomposite coatings were deposited on a Ti-modified stainless steel (D-9) substrate by pulsed DC magnetron sputtering. The main target was prepared with a composite target of Ta-Cu. The composition of Cu in TaN/Cu coating was controlled by increasing the surface area of the Cu target. The surface area of the Cu target was up to 5.21 cm^2^. Coatings of about 1.5-μm thickness were deposited using Ar for plasma and N_2_ as the sputtering reactive gas. The substrate temperature was 773 K. Further details about the deposition process and chemical composition of D-9 alloy substrate can be obtained from our previous report [21].

### 2.2. Physiochemical Characterization of TaN/Cu Coatings

The elemental composition of the TaN/Cu coating was analyzed using an electron probe microanalyzer. For elemental composition analysis, the TaN/Cu coating was prepared on a silicon substrate. The coating temperature and N_2_ flow rate were 773 K and 10 sccm, respectively. The X-ray diffraction (XRD) pattern was obtained using INEL XRD—3000 diffractometer(INEL, Celje, Slovenia). The glancing angle incidence was 5°. The radiation source was Cu Kα with λ = 1.54 Å. Surface morphology was observed using an XL30 ESEM Philips scanning electron microscope (SEM). Surface topography was examined using an atomic force microscope (AFM, Nanoscope E, digital Instruments Inc., USA). The images were collected in contact mode. A tungsten tip was used. The lateral resolution was about 0.1 nm.

### 2.3. Tribological Characterization of TaN/Cu Coatings

The nanohardness measurements were carried out using a nanoindenter (CSM, Switzerland) equipped with a Berkovich diamond indenter tip at a load of 10 mN. The D9 alloy substrates deposited with 2 µm thick TaN/Cu coatings were used for performing nanohardness analysis. The tribological behavior of the coatings was investigated using a commercial reciprocating tribo-tester (CETR UMT-2). Stainless steel balls with a diameter of 1 mm were used. All tests were performed at constant applied normal load of 10 mN and a sliding speed of 2 mm/s with a 2 mm stroke. The initial Hertizian contact pressure was 250 MPa. For each specimen, five replicate measurements were performed. The average value with the relative error of <5%, was used. The nanohardness measurements were carried out using a commercial ultra-nanohardness tester (CSM Switzerland) equipped with a Diamond Berkovich tip (load = 10 mN). The indentation depth was less than 1/10 of thickness of TaN/Cu coatings.

### 2.4. Evaluation of Antibacterial Activity of TaN/Cu Coatings

To mimic the natural environment, the TaN/Cu coated D-9 SS substrate was exposed to *P. aeruginosa*, isolated from freshwater biofilm. The isolated pathogen was identified using genus level biochemical analysis. To allow the bacteria to settle on the surface, 1 × 1 cm^2^ TaN/Cu coated D9 SS was exposed to *P. aeruginosa* cultured in a 10% nutrient bath for 24 h. Then, the substrate was gently withdrawn. The total viable cell count (TVC) on the biofilm grown on the TaN/Cu coating specimen was evaluated. The TVC, in terms of number of colony-forming unit (CFU), was evaluated using the Miles and Misra method. Then, the substrate was gently withdrawn and washed to remove the loosely adhered cells, if any. Using a sterile brush, the cells on the cultured substrate were stripped into 50 mL of sterile phosphate buffer, prepared by dissolving 0.0425 g KH_2_PO_4_ and 0.19 g MgCl_2_ in 1 L of water. For direct observation using an epifluorescence microscope (Nikon Eclipse E600; excitation filter BP 490; barrier filter O 515), the cultured substrates were subjected to gentle (sterile) water washing, dried in air, and flooded with a 0.1% aqueous solution of 0.1% acridine orange. To visualize the sessile bacterial population using SEM, the bacterial film formed on the substrates was chemically fixed using 2.5% aqueous glutaraldehyde at 4 °C for 1–4 days. After fixing, the traces of glutaraldehyde were removed by washing with water three times. Then, the substrates were dehydrated using graded ethanol (30–100%), air dried and desiccated. After gold–palladium (60:40) electrode coating, the samples were analyzed using SEM.

## 3. Results

### 3.1. Elemental Composition Analysis of TaN/Cu Nanocomposite Coatings

**Figure 1:**
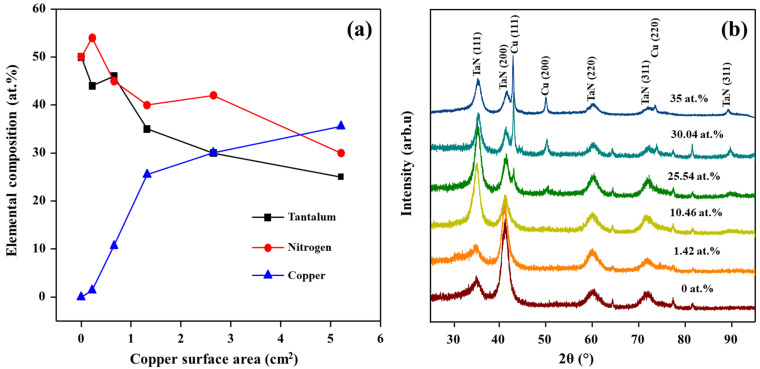
(**a**) Elemental composition of TaN/Cu coatings with respect to increases in the surface area of the Cu target; (**b**) XRD patterns of TaN/Cu coatings with different Cu content (at.% = Cu content in atomic %).

The chemical compositions of the TaN/Cu nanocomposite coatings deposited as a function of increasing the surface area of the Cu target are shown in Figure 1a. The chemical compositions and homogeneity of the TaN/Cu coatings were analyzed at ten different locations and average data was plotted. With respect to increases in the surface area of the Cu target, the TaN/Cu coatings with a Cu content of 1.42, 10.46, 25.54, 30.04, and 35.54 atomic % (at.%) were obtained. Moreover, these results showed two distinct regions. The first region corresponds to the lower Cu surface area of 0 to 0.22 cm^2^, where the coating content was rich in Ta compared to nitrogen and Cu. The second zone corresponds to the region where coatings were prepared with a Cu surface area of 2.65 to 5.21 cm^2^. In this region, the coatings were rich in Cu compared to Ta and nitrogen. Overall, it was determined that Ta–N bonds were partially replaced by Ta–Cu bonds with increasing Cu content [22].

### 3.2. Crystalline Structure of TaN/Cu Nanocomposite Coatings

The XRD patterns of pure TaN and TaN/Cu nanocomposite coatings containing 1.42, 10.46, 25.54, 30.04 and 35 at.% Cu are shown in Figure 1b. In all samples, the diffraction peaks of (111), (200), (220), (311) and (400) planes corresponding to the face-centered cubic (fcc) structure of TaN were observed (PCPDF file 03-2899) [23,24]. Coatings with very low Cu content (1.42 and 10.46 at.%) exhibited diffraction patterns similar to Cu-free TaN. No Cu phase was found in these samples, which implied that Cu atoms were trapped inside the TaN structure. An increase of Cu content above 10.46 at.% resulted in the formation of a bi-phasic structure of TaN and Cu. The intensities of TaN and Cu peaks were changed with increasing Cu content. The intensity of (200) peak was higher in the TaN/Cu coating with 1.42 at.% Cu. When the Cu content was increased further, the intensity of the TaN (111) peak was increased proportionally. Also, in the TaN/Cu coating with higher Cu content (25.54, 30.04 and 35 at.%), the intensity of the Cu peak became high and dominant when compared to the intensity of TaN peaks. The strongest Cu diffraction peak was detected at a 2θ value of 43.05°, corresponding to the (111) plane of metallic Cu (PCPDF file 03-065-2871).

### 3.3. Morphology of TaN/Cu Nanocomposite Coatings

**Figure 2:**
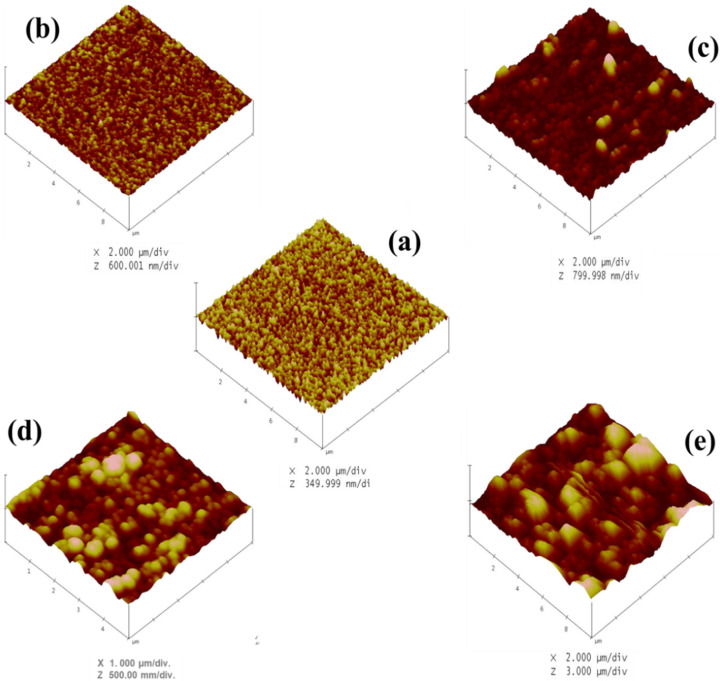
AFM images of TaN/Cu nanocomposite coatings with varying copper content (at.%): (**a**) 0, (**b**) 1.42, (**c**) 10, (**d**) 25.54 and (**e**) 35.

The surface topography of the TaN/Cu nanocomposite coatings with different Cu contents is shown in Figure 2a–e. The Cu content had a significant effect on the domain size. TaN/Cu coatings with 0, 1.42 and 10.46 at.% Cu exhibited a smooth granular structure, with individual grains having an average diameter ≥ 300 nm. Further increase in the content of Cu to 25.54 at.%, resulted in larger grains with diameters from 250 to 900 nm. Notably, until the Cu content reached 25.54 at.%, the shape of the grains remained circular. In contrast, in TaN/Cu coating containing 35 at.% Cu, the grain shape was changed from circular to random and the diameter/width was about 0.63 to 2.5 µm. The dark and bright areas indicate the variation in height of the domains. The gradual increase in height and size of the domain caused by the incorporation of Cu confirmed that Cu atoms served as the nucleation sites during the coating formation process.

**Figure 3:**
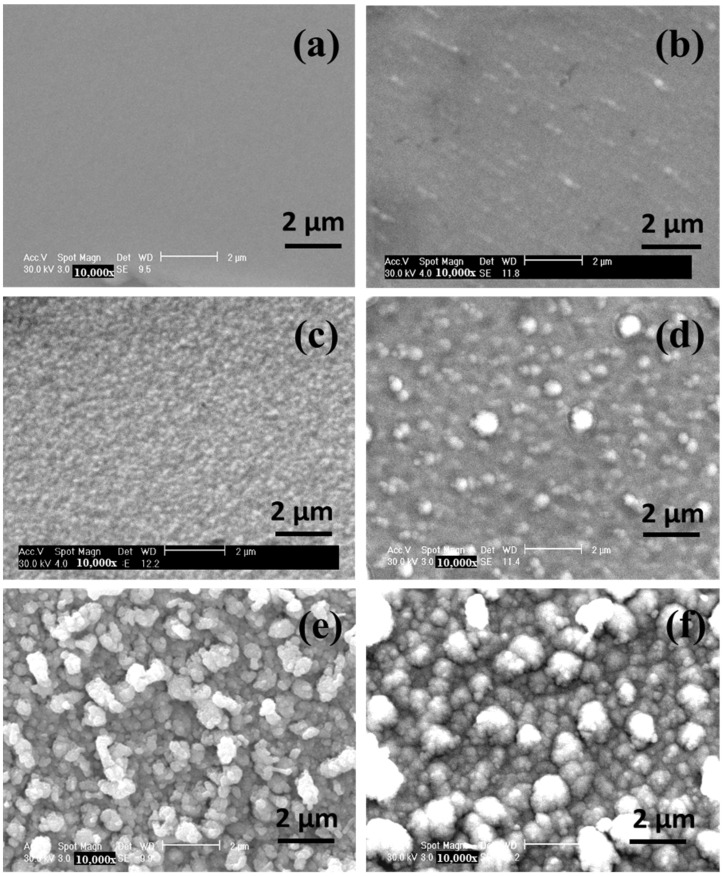
SEM image of TaN/Cu nanocomposite coatings with varying copper content (at.%): (**a**) 0, (**b**) 1.42, (**c**) 10.46, (**d**) 25.54, (**e**) 30.04, and (**f**) 35.

**Figure 4:**
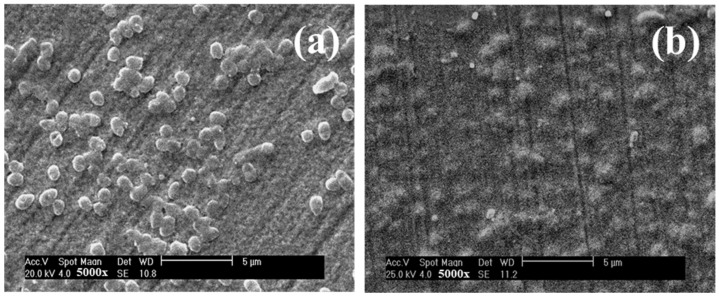
SEM images indicating the bacterial cell attachment in pure TaN coating (**a**), and TaN/Cu coating with a Cu content of 30.04 at.% (**b**).

The morphology of the TaN/Cu nanocomposite coatings was further examined using SEM (Figure 3a–f). The surface of pure TaN coatings was uniform (Figure 4a), without any structural formation, and this uniformity was altered upon the addition of 1.42 and 10.46 at.% Cu. Well defined particles were seen in the TaN coating with a Cu content of 25.54 at.% The dark spherical domains with a diameter of 200 nm and brighter domains having a diameter of 470 nm were observed. The population of brighter domains was considerably increased in coatings with a Cu content of 30.04 and 35 at.%. The diameter of larger domains was between 0.61 and 1.5 µm. The morphology of TaN/Cu were consistent with their XRD patterns and surface topography and confirmed the profound influence of Cu on the size and growth of TaN crystallites. The appearance of nanosized domains confirmed the formation of TaN/Cu nanocomposite coatings.

### 3.4. Antibacterial Properties of TaN/Cu Nanocomposite Coatings

**Figure 5:**
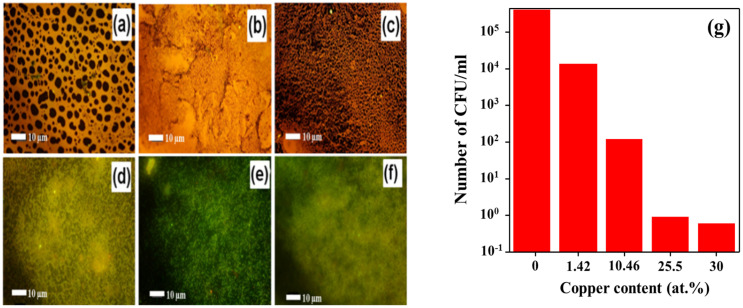
(**a**–**f**) Epifluorescence microscope images of TaN/Cu nanocomposite coatings with varying copper contents (at.%): (**a**) 0, (**b**) 1.42, (**c**) 10.46, (**d**) 25.54, (**e**) 30.04, and (**f**) 35. (**g**) Total viable count observed on TaN/Cu coatings with respect to changes in Cu content.

The effect of Cu on the antibacterial activity of TaN/Cu nanocomposite coatings deposited on a D-9 SS substrate with different Cu contents was systematically evaluated. Figure 4 shows the morphology of adherent bacteria observed by SEM after cell culture. Figure 4a shows the presence of a significant number of bacteria on the Cu-free TaN coating. On the other hand, Figure 4b shows that there are only a few bacteria on the TaN/Cu coating with a Cu content of 30.04 at.%. An epifluorescence microscope was used to provide further confirmation regarding the antibacterial properties of TaN/Cu coatings. Figure 5a shows an epifluorescence microscope image of the Cu-free TaN coating surface with a large number of live bacteria attached (orange fluorescence). The structures representing live bacteria cells seen on the Cu-free TaN surface could not be found in TaN/Cu coatings with Cu content of 1.42 and 10.46 at.%. However, the tone of orange fluorescence could be witnessed. At a Cu content of 25.54 at.%, the image of TaN/Cu coating appeared to be turning green, which indicates that a large portion of the bacteria were dead (Figure 5d–f). Orange fluorescence shows higher ribonucleic acid (RNA) concentration, indicating higher protein synthesis and suggests higher metabolic activity of active bacterial cells. Green fluorescence is corresponding to the natural deoxyribonucleic acid (DNA) in the cell. The dead bacteria cells confirmed the reduced metabolic activity and suggested that a certain level of stress was imposed on the cells.

Furthermore, to quantify the antibacterial properties of the coatings, the antibacterial activity of TaN/Cu nanocomposite coatings against *P. aeruginosa* was evaluated using the viable count method. As shown in Figure 5g, in the case of Cu-free TaN coating, the bacterial count was about 4 × 10^6^ CFU/mL. With the addition of Cu (10.46 at.%), this value dropped to 1.2 × 10^2^ CFU/mL, which is a decrease of more than 4 orders of magnitude when compared to Cu-free TaN coating. A further increase in the Cu content from 25.54 to 30.04 at.% resulted in a decrease in bacterial counts from 9 to 6 CFU/mL, respectively. These results confirmed that Cu is very effective in enhancing antibacterial activity. It has been reported that Cu^2+^ metal ions retain the ability to bind to nucleic acids to prevent the growth of bacteria [25]

### 3.5. Tribological Properties of TaN/Cu Nanocomposite Coatings

**Figure 6:**
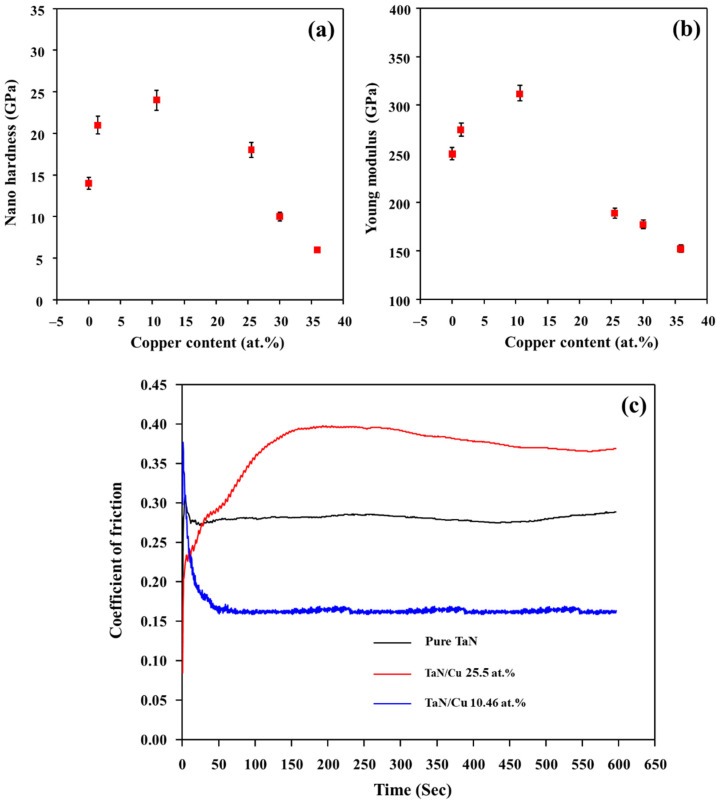
(**a**) Hardness, (**b**) young modulus of TaN/Cu nanocomposite coatings with respect to copper content (at.%), and (**c**) friction coefficient of coatings with respect to time (sliding distance).

For the practical application of TaN/Cu nanocomposite coating for antibacterial activity, the durability of the coating is important. The durability was assessed by investigating the basic mechanical and tribological properties of the coatings. The hardness and Young’s modulus of TaN/Cu nanocomposite coatings measured as a function of Cu content are shown in Figure 6a,b. An increased hardness of 20 GPa was obtained for the coating with a Cu content of 1.42 at.% compared to the Cu-free TaN coating, which had a hardness of 14 GPa. Based on preparation conditions, the hardness of single layer TaN coatings can be varied in the range of 14 to 26 GPa [26,27,28]. Cu-free TaN coating obtained here (14.7 GPa) is softer when compared to the maximum hardness reported for TaN coatings (26 GPa). At a Cu content of 10.46 at.%, hardness was further increased to GPa. When Cu content increased above 10.46 at.%, there was a steep decrease in hardness. At 35 at.% Cu content, hardness was decreased to 6 GPa.

In multi-phase metallic coatings, several factors including preparation conditions, grain size, coating thickness and elemental composition were considered as influencing the hardness [28,29]. Among them, the composition of soft phase elements such as, Ni, Cu, and Ag, had significant effect on hardness [30]. From the hardness results here, it is evident that the composition of Cu had a serious effect on the hardness. The changes in hardness of TaN/Cu coatings, noticed as a function of Cu content, can be compared with the effect of Cu content on the hardness of Al–Cu–N coatings prepared by Musil et al. using magnetron sputtering [31]. Al–Cu–N with 5 at.% Cu exhibited a hardness of 23 GPa. In the range of 7 to 12 at.% Cu content, the hardness was decreased to 18 GPa. A further increase in Cu content to 22.5 at.% decreased the hardness value to 6 GPa.

The mechanism behind the changes in hardness of TaN/Cu coatings observed as a function of increases in Cu content can be explained on the basis of a study on hardness of TaN coating by Bernoulli et al. [28]. In their study, a detailed analysis was performed regarding the phase, composition and hardness of TaN coatings prepared by varying N_2_/Ar flow ratios during deposition by magnetron sputtering. The trend in XRD peaks of TaN coatings provided valuable information about changes in hardness. The hardness of TaN coating with intense 35.8° and weak 41.6° peaks was higher (20.6 GPa) than the TaN coating with intense 41.6° and weak 35.8° peaks (16 GPa). XRD patterns of TaN coating (Figure 1b) with 0 (Cu-free) to 1.42 at.% Cu was similar to the TaN coating with lower hardness, where (200) peak is more intense than (111) peak. XRD patterns of TaN/Cu with Cu content from 10.46 to 35 at.% were similar to the TaN coating with higher hardness reported by Bernoulli et al. However, the notable difference is the appearance of the XRD peak (43.05°), corresponding to metallic Cu in TaN/Cu coatings with Cu content ≥25.54 at.%. This observation reveals the significant influence of Cu on the hardness of TaN/Cu coatings. Further, in the study of Bernoulli et al., the increase in hardness was attributed to a decrease in grain size [28]. The TaN coating with the highest hardness (20.6 GPa) had a grain size (calculated from the XRD pattern using the Debye–Scherrer formula) of 12.5 nm. The TaN coating with the lowest hardness had a grain size of 27.5 nm. The grain size of TaN with the highest hardness was 55% smaller than the grain size of the TaN coating that exhibited the lowest hardness.

Similarly, the grain size of TaN/Cu coating was as calculated on the basis of full width half maxima (FWHM) of (200) peak. The grain size of TaN/Cu with 0 (Cu-free), 1.42 and 10 at.% Cu was in the range of 4 to 3 nm. Whereas an increase in Cu from 25.54 to 35 at.% increased grain size from 5 to 8 nm. The grain size of TaN/Cu with 10.46 at.% Cu was 62% less than the TaN/Cu with 35 at.% Cu. The trend observed in XRD patterns and grain size discerned that though an increase in Cu composition induced the changes in lattice planes within the crystalline structure, it did not collapse the crystalline or ordered structure of TaN. The increase in the average size of TaN grains with increasing Cu content indicates that Cu atoms were serving as nucleation sites during the coating formation process. Therefore, changes in hardness of TaN/Cu coatings are not relevant to the existence of amorphous phase or crystalline-to-amorphous phase transformation. The diameter of circular TaN (200 nm) and random Cu domains (0.47 to 1.5 µm) observed in SEM images of TaN/Cu coatings (Figure 4) with up to 10.46 at.% and 25.56 to 35 at.%, reveals that the soft and larger Cu domains decreased the interaction between hard and smaller TaN crystallites, thereby decreasing the hardness.

The frictional characteristics of TaN and TaN/Cu coatings were evaluated by sliding a steel ball against the coating surface using a reciprocating tribo-tester under a constantly applied load. Average values of friction coefficients obtained for the pure TaN, TaN/Cu with 10.46 at.% and TaN/Cu with 25.54 at.% Cu are shown in Figure 6c. The friction coefficient of Cu-free TaN coating initially showed a value of around 0.3 and it decreased slightly to about 0.28 after a short sliding distance. In the case of the TaN/Cu with 10.46 at.% Cu, the friction coefficient dropped to about 0.16 from the initial value of about 0.35 as the sliding distance increased. However, with 25.54 at.% Cu, the TaN/Cu composite coating showed a relatively high friction coefficient of about 0.38.

**Figure 7:**
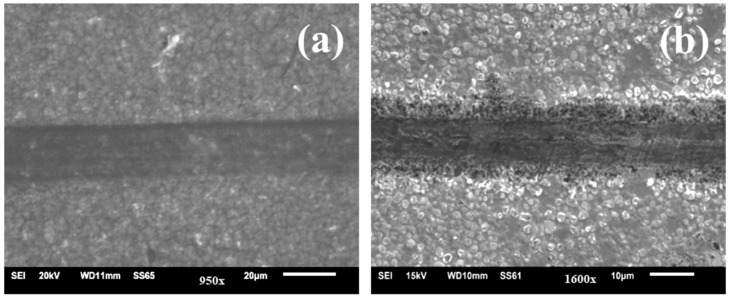
SEM image of the wear track of TaN/Cu nanocomposite coating with (**a**) 10.46 at.% and (**b**) 25.54 at.% copper content.

Figure 7a,b shows the SEM images of the wear track obtained after the friction tests for the two TaN/Cu composite coatings. It was clearly evident that the TaN/Cu with 25.54 at.% Cu experienced more surface damage than the TaN/Cu with 10.46 at.% (Figure 7a). The low frictional characteristics of the TaN/Cu with 10.46 at.% can be beneficial. On the other hand, TaN/Cu with 25.54 at.% Cu exhibited relatively high frictional force and led to the generation of high interfacial shear stress and caused the removal of coating from the SS substrate [32,33,34,35]. These results were consistent with the hardness data shown in Figure 6a, where the hardness of TaN/Cu with 10.46 at.% Cu was significantly higher than that of the TaN/Cu with 25 at.% Cu. As noticed in the multi-metal interface system, stainless steel—tungsten carbide brazed with AgCuTi and AgCuIn alloys, reported by Yan et al. [36], incorporation of In element exhibited greater alloying properties and rendered a more corrosion resistant and mechanically stable joint than Ti. In view of intermetallic bonding, an increase in the quantity of Cu in TaN/Cu decreased interfacial integration with the stainless steel (D9) substrate, therefore, TaN/Cu coating with 10.46 at.% Cu was more adherent than Tan/Cu with 25 at.% Cu.

## 4. Conclusions

The chemical composition, structural, morphological, antibacterial and tribological properties of Cu-free TaN and TaN/Cu nanocomposite coatings on D-9 SS substrate were investigated. XRD studies confirmed the formation of TaN and Cu bi-phasic structures in TaN/Cu coatings. Increases in Cu content decreased the coarsening of TaN crystallites. SEM and AFM images visualized the well-defined morphological change caused by the incorporation of Cu. The surface of Cu-free TaN was smoother, while TaN/Cu coatings were observed to be rough. The antibacterial activity against *P. aeruginosa* was improved with increasing Cu content, as confirmed by the total viable cell count. The bacterial count dropped by more than 4 orders of magnitude with a Cu content of 10.46 at.%, compared to Cu-free TaN coating. TaN/Cu with Cu content of 10.46 at.% exhibited a peak hardness of 24 GPa and a Young’s modulus of 295 GPa. The frictional properties, as well as the durability of the coating with a Cu content of 10.46 at.%, was better than those with a Cu content of 25.54 at.%. In consideration of both the antibacterial activity and durability, TaN/Cu nanocomposite coating with a Cu content of 10.46 at. % was considered as having the potential for practical applications.

## Data Availability

Upon reasonable request, the data supporting this investigation are available from the corresponding authors.
